# One Size Does Not Fit All: Medication Reconciliation and Review at the Hospital at Home

**DOI:** 10.7759/cureus.47419

**Published:** 2023-10-21

**Authors:** Daniela Madeira, Flávia Baduy, Ana Orfão, Clara Matos, Rui Osório, Ana C Brito

**Affiliations:** 1 Internal Medicine, Hospital Professor Doutor Fernando Fonseca, Amadora, PRT; 2 Family Medicine, Unidade de Saúde Familiar Tejo, Agrupamento de Centros de Saúde (ACES) Loures/Odivelas, Loures, PRT; 3 Hospital at Home, Hospital Professor Doutor Fernando Fonseca, Amadora, PRT

**Keywords:** hospital at home, deprescription, polypharmacy, medication revision, medication reconciliation

## Abstract

Mistakes in the medication process are frequent and a common cause of morbidity and mortality. Medication reconciliation (MRec) and medication review (MRev) are the processes of creating the most accurate medication list and adapting it to optimize the effectiveness of medicines and minimize adverse effects. This is crucial in all stages of medical care, especially at discharge. The present study aims to evaluate and describe the process of MRec and MRev, with a focus on deprescribing, that we conduct at the Hospital at Home.

We performed a retrospective cohort study including adult patients admitted at our Hospital at Home from 1 November 2022 to 30 April 2023. MRec and MRev were applied during hospitalization, according to patients’ characteristics and clinical evolution, and then communicated to patients upon discharge.

Our study involved 125 patients, with an average age of 67.6±18.0 years, and half of them had polypharmacy. We discovered discrepancies in 43.2% of patient’s medication and did deprescribing in one-third of them. In the deprescribing group, patients were significantly older (mean age, 76.1 versus 66.4 years; p=0.044).

It is imperative to create mechanisms to identify patients at a greater risk of adverse drug events and to minimize the burden of care and harms associated with treatments. The Hospital at Home could be an opportunity, although further research is essential.

## Introduction

Taking medicines is crucial for preventing and treating illnesses, but taking them incorrectly can cause significant harm, such as a negative impact on the quality of life, and threaten the safety of patient care [1,2]. Mistakes in the medication process are a common cause of inhospital morbidity and mortality and a leading contributor to preventable harm in healthcare systems worldwide [1,3]. Globally, these errors have an estimated annual cost of US$42 billion [1]. Discharge from the hospital is one of the most crucial steps since medications may have been changed, new illnesses may have been diagnosed, and different healthcare providers may be involved in their care [4,5]. According to research, 66% of adverse events within three weeks after hospital discharge are due to adverse drug events, and 62% of these events can be prevented [6]. During this stage of medical care, common errors include incorrect medication continuation or discontinuation, duplication errors, and incorrect dosing, which can lead to serious consequences such as death, emergency department visits, and unplanned hospitalizations [7,8]. To ensure patient safety, it is essential to promote and enhance medication systems, including ordering, prescription, preparation, dispensing, administration, and monitoring, as well as empowering patients with health literacy [1,9].

The medication reconciliation (MRec) concept was first described in 2003 and is now considered a crucial part of safe medication use and good clinical care by both the Joint Commission and the World Health Organization [3,10,11]. MRec is defined as the process of creating the most accurate list possible of a patient’s medications, including drug name, dosage, frequency, and route, and comparing it against current and planned prescriptions [4,12]. Decisions are then made on whether medications should be continued, changed, or discontinued, accordingly to patients’ and carer’s views, the safety of each drug, and compliance. This process is known as medication review (MRev) [4,7,9]. The goal is to identify discrepancies, document changes, and provide patients with the correct medications across all healthcare interfaces to optimize the effectiveness of medicines, minimize medication-related issues, and reduce waste [4,7,9]. This is a complex process that requires multiple steps, data sources, and the efforts of several people [4,10,13]. All of these procedures should undeniably be patient-centered by involving their preferences in treatment decisions through shared decision-making [7]. Deprescribing is the process of gradually decreasing the number and dosage of drugs that may be harmful or no longer needed, to reduce medication burden, avoid adverse effects, and improve outcomes while maintaining or improving the quality of life [14,15]. This is a vital aspect of managing chronic conditions and should be considered in MRev [15,16].

One of the main challenges faced by MRec is the communication gap among patients, caregivers, and healthcare professionals, so physical proximity can facilitate the sharing of information [17]. The present study aims to evaluate and describe the process of MRec and MRev, focusing on deprescribing, that we conduct at the Hospital at Home, located at the Hospital Professor Doutor Fernando Fonseca, Portugal, in the transition of care from the hospital to our unit and then to discharge. Hospital at Home is a clinical service that provides acute hospital-level care to selected individuals in their homes or nursing homes, substituting for acute inpatient hospital care. This service includes staff, equipment, technologies, medication, and skills that are typically provided in hospitals [18,19].

## Materials and methods

Population and study design

We performed a retrospective cohort study and collected data from adult patients who were admitted at our Hospital at Home in order to explore the characteristics associated with medication review, particularly deprescription.

We used universal sampling and convenience, so all patients aged 18 years old or older who were admitted to our Hospital at Home from 1 November 2022 to 30 April 2023 were included. Exclusion criteria included patients who were readmitted to the hospital during home hospitalization; however, there were no readmissions in this period.

Demographic and clinical data were gathered by reviewing the electronic data of patients. We utilized the Charlson comorbidity index (CCI) score to evaluate the severity of patients’ medical conditions [20].

This study was conducted in compliance with the Declaration of Helsinki, International Council for Harmonisation of Technical Requirements for Pharmaceuticals for Human Use (ICH) Good Clinical Practice, and all applicable laws and regulations.

Our primary goals were to characterize the process of medication review applied to our population and to identify patient characteristics associated with deprescription.

Assessment tools to support medication review

MRec and MRev were used at the first home visit, collecting the current medication list, by asking the patient and caregivers, looking up for boxes and bags of medication in the home (including herbal medicines), and consulting individual files. Subsequently, any necessary changes to the patient’s therapy were made during hospitalization, considering all the information collected, and then communicated and explained to both the patient and their family upon discharge. All the changes were described in patients’ process file. We utilized clinical criteria and patient evaluation during hospitalization to decide on medication adjustments. In older adults (≥65 years old), we used the Screening Tool of Older Persons’ Prescriptions (STOPP) and the Screening Tool to Alert to Right Treatment (START) to review potentially inappropriate medications [21]. We defined polymedication as taking five or more medications [22].

Statistical analysis

Continuous data are presented as the mean±standard deviation (SD). Binary and categorical variables are expressed as absolute and relative frequencies. For the comparison of means, the Student t-test was used. For the comparison of proportions, the chi-square test and Fisher’s exact test were used, whenever applicable. Multivariate analysis was performed using multiple logistic regression models. Data analysis was performed using the Stata® software (version 16) (StataCorp LLC, College Station, TX). The significance level was defined at 0.05.

## Results

Demographics and clinical characteristics of the study population

A total of 125 patients were included in the study. Patient characteristics are described in Table 1.

**Table 1:** Baseline characteristics of participants Continuous variables are presented as mean±standard deviation; binary variables are presented as frequency (%)

Total	n=125
Age (years)	67.6 (±18.0)
Female	64 (51.2%)
Charlson comorbidity index	4.1 (±2.8)
Residence	
Home	108 (86.4%)
Nursing home	17 (13.6%)
Polypharmacy	63 (50.4%)

The majority of patients lived at home (86.4%), while the remaining resided in a nursing home. As expected, residents in nursing homes were significantly older when compared to patients living at home (mean age, 85.8 versus 64.7 years; p<0.001). Patients in nursing homes, when compared to those living at home, had significantly higher CCI scores (CCI mean score of 6.2 versus 3.8, respectively) and were more likely to be polymedicated (82% versus 45.4%, respectively); however, after adjusting to age, these associations were no longer significant.

Half of our patients were polymedicated. These patients were also older than non-polymedicated (77.2 versus 57.9 years; p<0.001) and had a significantly higher CCI (5.6 versus 2.7; p<0.001).

As expected, we also found a moderate positive correlation between age and the CCI (r^2^=0.542; p<0.001).

Description of medication alterations in the study population

We found discrepancies and adjusted medication lists of 54 (43.2%) patients. Additionally, 29 patients began taking new medications, while 19 stopped taking some drugs. Table 2 provides details concerning drug adjustments. Antihypertensive drugs were the most altered drugs (n=27), followed by anticoagulants and antidiabetic drugs (both n=6). We started more drugs in younger patients and with lower CCI. Our results showed that the number of alterations on medications occurred regardless of where the patient lived (home or nursing home) (43.5% versus 41.2%; p=0.852).

**Table 2:** Drug changes

	Total	Suspended	Prescribed	Switched	Frequency changed	Dose changed
Patients, n (%)	54 (43.2%)	19 (15.2%)	29 (23.2%)	6 (4.8%)	2 (1.6%)	11 (6.4%)
Drugs (n)						
Antihypertensive	27	9	11	2	2	5
Anticoagulant	6	1	3	1	0	1
Antidiabetic	6	2	1	2	0	1
Folic acid	4	0	4	0	0	0
Antiplatelet	3	2	1	0	0	0
Diuretic	3	0	3	0	0	0
Antiarrhythmic	2	2	0	0	0	0
Drugs for hyperuricemia	2	1	1	0	0	0
Opioids	2	1	0	1	0	0
Proton pump inhibitor	2	0	2	0	0	0
Statin	2	2	0	0	0	0
Antidepressive	1	0	1	0	0	0
Antipsychotic	1	0	1	0	0	0
Benzodiazepine	1	1	0	0	0	0
Bronchodilator	1	0	1	0	0	0
Insulin	1	0	0	0	1	0
Pregabalin	1	0	1	0	0	0
Others	3	2	1	0	0	0

Analysis of deprescription in the study population

In 12.8% of patients, we deprescribed certain drugs that posed a greater risk of harm than benefit to the patients. Patients subjected to deprescription were found to be significantly older compared to others (mean age, 76.1 versus 66.4 years; p=0.044). There were no significant associations between deprescription and other explanatory variables such as polypharmacy, CCI, or residency, after adjustment for age.

## Discussion

MRec at discharge seems to be one of the most important interventions to reduce errors at home after hospital admission, such as good communication with patients and families [8]. Hundreds of organizations have shown that the inaccurate communication of medical information at transition points is responsible for as many as 50% of all medication errors and up to 20% of adverse drug events in the hospital setting [23]. We found medication discrepancies in almost half of patients, and one-third of them were medicated with drugs of no benefit. Therefore, in our unit, we focus our approach on MRec and MRev to optimize all medication procedures and reduce adverse effects and, therefore, empower our patients with clear and as simple as possible explanations about the therapy instituted. Being close to the patient, caregivers, and home enables us to access more reliable information and facilitates communication, the development of individualized strategies, and the clarification of doubts.

In our study population, increasing age was associated with polypharmacy, residency in nursing homes, and a higher CCI score. Patients who were subjected to deprescribing were also significantly older compared to those whose medication was not simplified. Although we expected that, as our population grows older, becomes more dependent, and develops more comorbidities, they might need more medications, we also know that this type of population has a higher frailty index [7]. This augments the probability of inappropriate prescriptions, incidents of adverse drug reactions (which are usually more severe in older people), increased morbidity and mortality, and elevated costs [2,7]. Therefore, it is crucial to conduct MRec and MRev and always check with patient and their caregivers about their pharmacologic therapy (especially dosages and frequency), particularly in this group of patients.

Regarding drug changes, in elderly patients, we mainly have suspended drugs used to treat chronic diseases that aim to improve prognosis and decrease long-term complications, such as antihypertensives, antidiabetics, and statins. The drugs we prescribed, including antidepressants, antipsychotics, proton pump inhibitors, and pregabalin, were intended to improve symptomatic control and patients’ quality of life. This aligns with the primary goals of MRec and MRev, which prioritize the safety and well-being of the patient.

One of the limitations of our study pertains to the relatively small sample size and to the small representation of patients residing in nursing homes, who might benefit even more from medication review and deprescription [24].

## Conclusions

MRec and MRev constitute a particular challenge in medication management and require multiple resources. In the Hospital at Home, it is easier to obtain accurate and reliable information and establish communication with patients and families. This is an opportunity to minimize medication discrepancies and subsequent adverse drug events.

The high amount of patients with drug inconsistencies, particularly the elderly with polypharmacy and multiple comorbidities, showed us that it is imperative to create mechanisms to identify patients at a greater risk of drug iatrogenesis in order to allocate more efforts to them and to reduce the burden of care and harm associated with treatments. Further research is necessary to understand the processes of MRec, MRev, and deprescribing, as well as their impacts and medication cascade. Additionally, evaluating other outcomes such as mortality, readmissions, and emergency department visits is crucial.
